# Effects of a single night of continuous positive airway pressure on spontaneous brain activity in severe obstructive sleep apnea

**DOI:** 10.1038/s41598-023-36206-0

**Published:** 2023-06-02

**Authors:** Yuanfeng Sun, Fei Lei, Lian Luo, Ke Zou, Xiangdong Tang

**Affiliations:** Sleep Medicine Center, West China Hospital, Sichuan University, Chengdu, China

**Keywords:** Sleep disorders, Circadian rhythms and sleep

## Abstract

This study aimed to investigate the effect of a single night of continuous positive airway pressure (CPAP) treatment on spontaneous brain activity and the underlying neuropathological mechanisms in patients with severe obstructive sleep apnea (OSA). The study involved 30 severe OSA patients and 19 healthy controls (HC). Fractional amplitude of low-frequency fluctuation (fALFF) and regional homogeneity (ReHo) methods were employed to evaluate spontaneous brain activity in all participants. Following a single night of CPAP treatment, ReHo values increased in the bilateral caudate and decreased in the right superior frontal gyrus. The fALFF values increased in the left orbital part of the middle frontal gyrus and the right orbital of the inferior frontal gyrus (Frontal_Inf_Orb_R). However, fALFF values decreased in the medial part of the left superior frontal gyrus and the right supramarginal part of the inferior parietal lobe. Pearson correlation analysis revealed a positive relationship between the change in the fALFF in the Frontal_Inf_Orb_R and the change in REM sleep duration (*r *= 0.437, *p* = 0.016) following a single night of CPAP treatment. We concluded that observing changes in abnormal fALFF and ReHo in OSA patients before and after a single night of CPAP treatment may enhance our understanding of the neurological mechanisms in patients with severe OSA.

## Introduction

Obstructive sleep apnea (OSA) is a common chronic sleep-related breathing disorder characterized by partial or complete closure of the respiratory tract during sleep. This leads to recurrent intermittent hypoxia, carbon dioxide retention, and sleep fragmentation^1^. The prevalence of OSA is higher than previously estimated, with moderate to severe OSA affecting approximately 20% of adult men and 10% of postmenopausal women^2^. A study involving 38,000 Russian citizens aged 30–70 years revealed that 48.9% of participants exhibited an apnea–hypopnea index (AHI) ≥ 5, 18.1% had an AHI ≥ 15%, and 4.5% an AHI ≥ 30^3^. The disease can result in serious health complications, such as heart disease, high blood pressure, gastroesophageal reflux, and other disorders^4^. However, the specific mechanism underlying brain dysfunction in OSA patients remains unclear.

Continuous positive airway pressure (CPAP) is the first-line treatment for patients with moderate to severe OSA. Following just one night of CPAP treatment, intermittent hypoxia can be rapidly corrected, sleep fragmentation significantly reduced, and the proportion of slow-wave and REM sleep markedly increased. In some severe OSA patients, daytime sleepiness disappeared and their mental state improved significantly the following day after a single night of CPAP treatment. Previous studies have demonstrated that one month of CPAP treatment can effectively reverse the compensatory response of the bilateral cerebellar posterior lobe and functional network damage caused by OSA^5^. White matter integrity can also be restored in several brain regions after 12 months of CPAP treatment^6^. However, there have been limited studies on the effects of single-night CPAP treatment on brain function in OSA patients. Consequently, we hypothesize that a single night CPAP of treatment may also significantly improve brain function.

To gain a deeper understanding of the pathophysiological and neuroimaging mechanisms of OSA, resting-state functional magnetic resonance imaging (rs-fMRI) is an important method to track hemodynamic changes in the brain using changes in MRI signal intensity. As a critical rs-fMRI measure, the fractional amplitude of low-frequency fluctuation (fALFF) approach, which is the ratio of power spectrum of low frequency (0.01–0.08 Hz) to that of the entire frequency range (0–0.25 Hz), may effectively suppress non-specific signal components in the rs-fMRI, and therefore would significantly improve the sensitivity and specificity in detecting regional spontaneous brain activity^7^.

Regional homogeneity (ReHo) is another newly developed method to evaluate the similarity or consistency of spontaneous low-frequency blood oxygenation level-dependent (BOLD) signal fluctuations within a region in the whole brain voxel analysis^8^. The ReHo of BOLD activity is now thought to account for differences in neurovascular coupling and task activation. The ReHo method has also been successfully used to study the functional regulation of patients in the resting state, reflecting the changes in temporal neuronal activity in specific regions. If ReHo increases, the local connections of neurons in local brain regions are enhanced. However, the reduced ReHo indicates weakened local connections of local neurons. Therefore, it can be concluded that there is a significant correlation between abnormal ReHo and changes in neuronal activity in local brain functional regions. In other words, when the ReHo is abnormal, the synchronous activity of local neurons changes. Only one study has reported significant changes in ReHo in the bilateral middle temporal gyrus, medial frontal gyrus, supplementary motor area, and left superior frontal gyrus after one month of CPAP treatment in OSA^9^. In our previous study, we reported that amplitude of low-frequency fluctuation (ALFF) changes after a single night of CPAP treatment in severe OSA patients^10^. The fALFF is a different evaluation method from ALFF, which has a better inhibitory effect on non-specific signals in resting state fMRI. However, there have been no studies of fALFF in patients with OSA before and after CPAP treatment.

Based on the aforementioned consideration, we hypothesized that OSA patients exhibit abnormalities in brain activity, and that these abnormalities could be partially reversed following a single night CPAP treatment. Through this study, we aimed to utilize imaging evidence to gain a deeper understanding of the impact of OSA of brain function and the onset and progression of the disease. To test this hypothesis, we assessed the disparity in fALFF/ReHo values between a healthy control (HC) and OSA patients. Additionally, we investigated changes in spontaneous brain activity in OSA patients after a single night of CPAP therapy. Furthermore, we examined the correlation between the alterations in spontaneous brain activity and polysomnographic data.

## Results

### Clinical characteristics

The demographic and polysomnography data are shown in Table 1. There were no significant differences in age, education, sleep latency, time in bed, total sleep time (TST), sleep efficiency, and the time of REM sleep between the OSA patients and HC groups.

**Table 1:** Demographic and polysomnographic data of participants.

	Pre-CPAP (n = 30)	Post-CPAP (n = 30)	HC (n = 19)	P^a^	P^b^
ESS	15.4 ± 6.0	15.4 ± 6.0	3.4 ± 1.5	0.906	ns
Education (years)^§^	15.0 (12.0, 16.0)	15.0 (12.0, 16.0)	12 (12, 19)	0.566	ns
Age (year)	42.5 ± 5.8	42.5 ± 5.8	40.3 ± 2.9	0.361	ns
Sleep latency (min)	9.2 ± 14.0	12.0 ± 16.2	12.0 ± 7.7	0.465	0.369
Time in bed (min)	508.8 ± 54.1	495.7 ± 48.9	489.9 ± 53.6	0.331	0.241
Total sleep time (min)	452.7 ± 67.4	429.4 ± 51.8	418.4 ± 55.2	0.142	0.06
Sleep efficiency (%)	88.8 ± 7.7	86.8 ± 8.1	85.8 ± 9.9	0.345	0.279
REM (min)	63.9 ± 33.6	111.0 ± 36.2	73.4 ± 23.0	< 0.001	0.04
N1 (min)	230.4 ± 111.1	64.1 ± 33.1	81.8 ± 42.3	< 0.001	< 0.001
N2 (min)	152.2 ± 74.9	222.1 ± 46.1	246.9 ± 53.0	< 0.001	< 0.001
N3 (min)§	0 (0, 4.0)	11.0 (0.5,55.1)	11.5 (0.5, 29.0)	0.002	0.036
R/TST (%)	14.2 ± 6.6	25.5 ± 6.3	17.4 ± 4.5	< 0.001	0.05
N1/TST (%)	50.9 ± 21.6	14.9 ± 7.2	19.8 ± 10.3	< 0.001	< 0.001
N2/TST (%)	33.6 ± 17.1	52.0 ± 10.7	59.2 ± 10.1	< 0.001	< 0.001
N3/TST (%)^§^	0 (0, 0.8)	3.0 (0.1, 13.1)	2.6 (0.1, 6.6)	0.002	0.027
AHI(n/h)	71.0 ± 21.1	13.6 ± 8.6	3.5 ± 1.6	< 0.001	< 0.001
T90 NREM (%)^§^	46.9 (16.2, 64.7)	2.9 (0.1, 11.0)	0 (0, 0.6)	< 0.001	< 0.001
T90 REM (%) ^§^	60.4 (40.4, 77.5)	0.3 (0, 2.6)	0 (0, 2.1)	< 0.001	< 0.001
T90 TST (%)^§^	49.5 (20.1, 68.9)	2.5 (0.2, 8.6)	0 (0, 0.8)	< 0.001	< 0.001
SaO2 TST (%)	87.5 ± 5.0	94.3 ± 1.4	94.5 ± 1.6	< 0.001	< 0.001

^§^Nonparametric test (Mann–Whitney U). P^a^, test between pre-CPAP OSA patients and HC; P^b^, test between CPAP night and pre-CPAP OSA patients. REM, the time of REM stage sleep; SWS, slow wave time; ESS, Epworth sleepiness scale; T90 NREM: the percent sleep time below 90% SaO_2_ in NREM sleep stage; T90 REM: the percent sleep time below 90% SaO_2_ in REM sleep stage; T90 TST: the percent sleep time below 90% SaO_2_ in total sleep time; ns, no statistically significant.

The OSA patients showed significantly higher, Epworth Sleepiness Scale (ESS), AHI and the time of N1 sleep, but lower mean in SaO_2_ of total sleep time, the percent sleep time below 90% SaO_2_ (T90) in REM, NREM stage and total sleep time, compared with HC. After one-night CPAP treatment, the time of REM sleep, N3 sleep, the mean SaO_2_ of total sleep time increased significantly. AHI and the percent sleep time below 90% SaO_2_ (T90) in REM, NREM stage and total sleep time decreased significantly.

### ReHo and fALFF analyses

**Figure 1:**
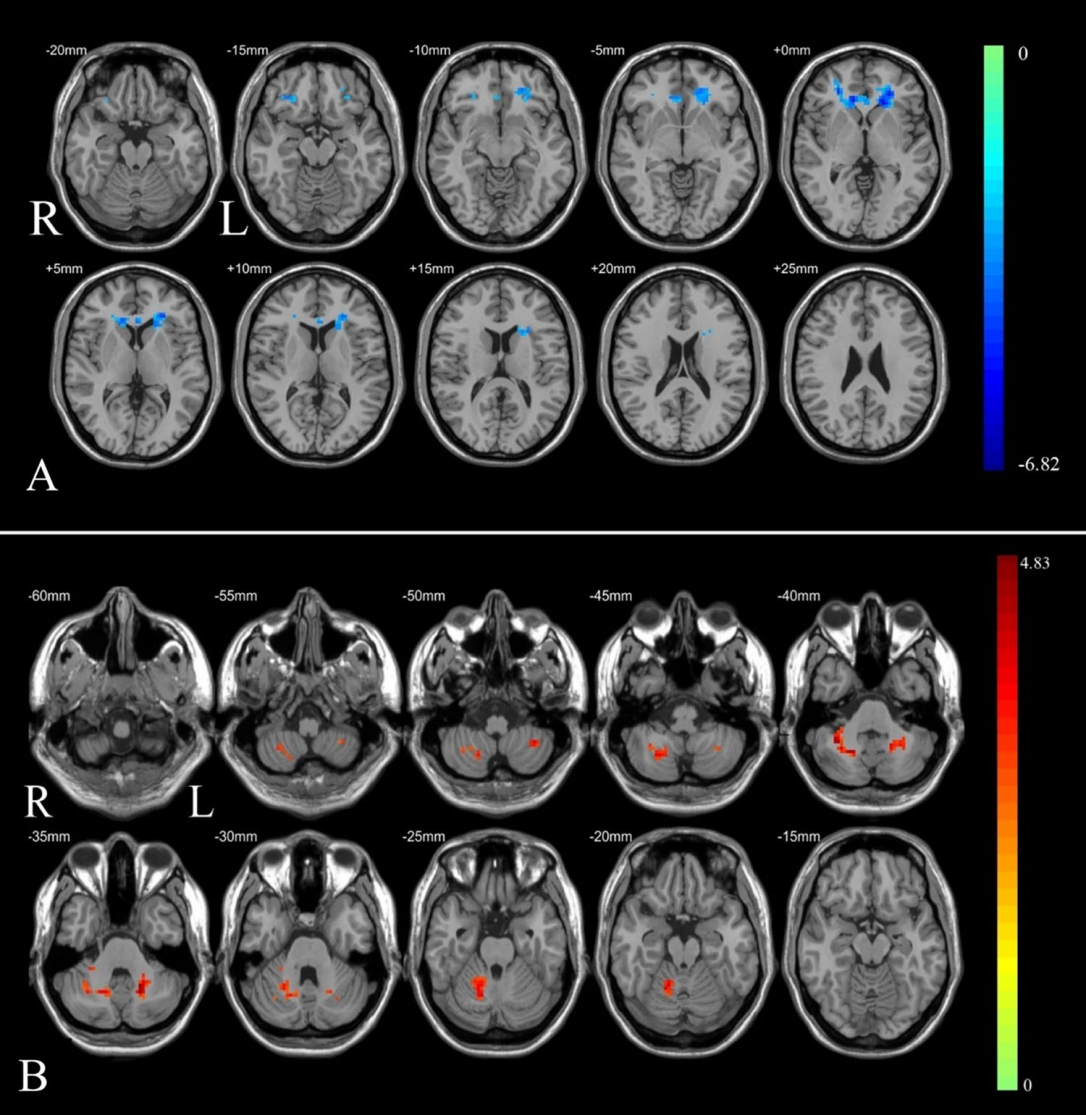
Brain regions comparisons between OSA patients and HC. (**A**) ReHo values showed a significant decrease in the bilateral caudate in OSA patients. (**B**) The value of fALFF increased in the bilateral cerebellar region 8 in OSA patients. All clusters were reported with a voxel-level threshold of *P* < 0.001, GRF correction, and cluster-level of *P* < 0.05, two tailed.

Compared to the HC groups, all patients showed a significant decrease in ReHo values in the bilateral caudate. The value of fALFF increased in the bilateral cerebellar region 8. These results were displayed in Table 2 and Fig. 1.

**Table 2:** Differences of fALFF/ReHo in brain regions between OSA patients and HC.

	Brain area	Brodmann area	Clusters (voxels)	Clusters (mm^3^)	MNI coordinates			Peak intensity
					X	Y	Z	
fALFF								
Pre-CPAP versus Health Control								
Pre-CPAP > HC	Cerebelum_8_R	none	177	4779	33	− 51	− 39	4.50891
	Cerebelum_8_L	none	62	1674	− 18	− 60	− 33	4.82559
ReHo								
Pre-CPAP versus Health Control								
Pre-CPAP < HC	Caudate_L	BA47_L	208	5616	− 18	27	3	− 5.03806
	Caudate_R	BA11_R	145	3915	26	52	25	− 5.24238

All clusters were reported with a voxel-level threshold of *P* < 0.001, Gaussian random field (GRF) correction, and cluster-level of *P* < 0.05, two tailed.

**Figure 2:**
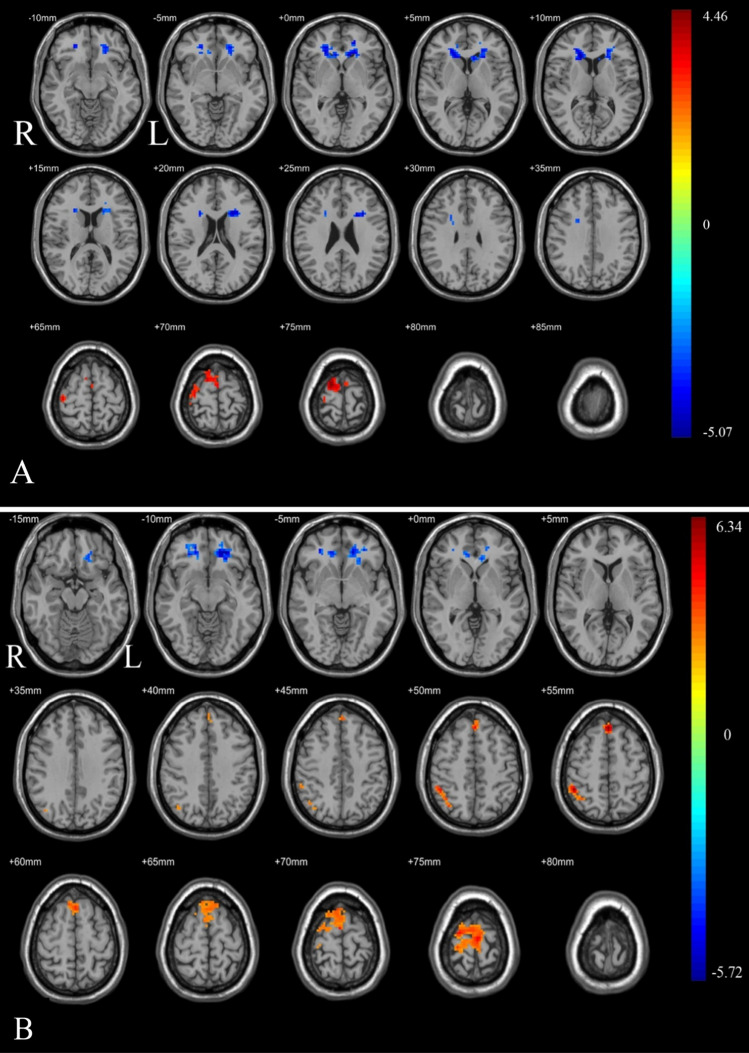
Brain regions comparisons of OSA patients before and after CPAP treatment (**A**) ReHo values increased in the bilateral caudate and decreased in the Frontal_Sup_R after a single night CPAP treatment. (**B**) fALFF values increased in the Frontal_Mid_Orb_L and Frontal_Inf_Orb_R, but decreased in the Frontal_Sup_Medial_L and Parietal_Inf_R after a single night of CPAP treatment. All clusters were reported with a voxel-level threshold of *P* < 0.01, GRF correction, and cluster-level of *P* < 0.05, two tailed.

After a single night of CPAP treatment, the ReHo values increased in the bilateral caudate and decreased in the right superior frontal gyrus (Frontal_Sup_R). The fALFF values in the left orbital part of the middle frontal gyrus (Frontal_Mid_Orb_L) and right orbital of the inferior frontal gyrus (Frontal_Inf_Orb_R) increased, but the fALFF values in the medial part of the left superior frontal gyrus (Frontal_Sup_Medial_L) and right supramarginal part of the inferior parietal lobe (Parietal_Inf_R) decreased. These results were presented in Table 3 and Fig. 2. Clusters with significant changes in fALFF/ReHo values are extracted as Regions of Interest (ROIs) for further analyzed.

**Table3:** Differences of fALFF/ReHo in brain regions between pre-CPAP and post-CPAP.

	Brain area	Brodmann area	Clusters (voxels)	Clusters (mm^3^)	MNI coordinates			Peak intensity
					X	Y	Z	
fALFF								
Pre-CPAP versus post-CPAP								
Pre-CPAP < post-CPAP	Frontal_Mid_Orb_L	BA11_L	129	3483	− 24	39	− 9	− 5.71776
	Frontal_Inf_Orb_R	BA47_R	74	1998	30	42	− 6	− 4.75524
Pre-CPAP > post-CPAP	Frontal_Sup_Medial_L	BA6_R	473	12,771	0	33	54	6.22289
	Parietal_Inf_R	BA40_R	79	2133	48	− 45	54	5.97152
ReHo								
Pre-CPAP versus post-CPAP								
Pre-CPAP < post-CPAP	Caudate_L	BA25_L	284	7668	− 11	22	6	− 4.70186
	Caudate_R	BA47_R	207	5589	18	24	3	− 5.07345

All clusters were reported with a voxel-level threshold of *P* < 0.01, GRF correction, and cluster-level of *P* < 0.05, two tailed.

### Correlation analysis of fALFF/ReHo and polysomnography parameters

**Figure 3:**
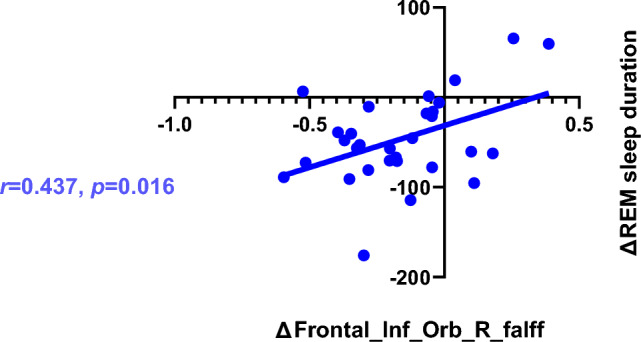
Correlation analysis between the changes of fALFF values and the changes in polysomnographic parameters.

Based on the whole-brain ReHo and fALFF analysis, six ROIs were identified. The Montreal Neurological Institute (MNI) coordinates of these ROIs were obtained from Table 3, and the radius of each ROI was set to 5 mm. The mean values of the ROIs based on ReHo were not significantly correlated with polysomnographic parameters. However, the change of the fALFF (the fALFF value before CPAP treatment subtracted from the value after CPAP) in the Frontal_Inf_Orb_R was positively correlated with the change of REM sleep duration (the REM sleep stage duration before CPAP treatment subtracted from the duration after CPAP) (*r* = 0.437, *p* = 0.016). The results were shown in Fig. 3.

## Discussions

In this study, we analyzed fALFF and ReHo brain activity in patients with OSA and investigated changes in the associated values following a single night of CPAP treatment. There were found to be significant differences in ReHo in the bilateral caudate nucleus and fALFF in the bilateral cerebellum 8 lobes. After a single night of CPAP treatment, multiple brain regions exhibited alterations in fALFF and ReHo. Furthermore, the results of the Pearson correlation analysis indicated that changes in fALFF in the frontal lobe were associated with changes in REM sleep duration after one-night CPAP treatment.

The decrease in bilateral caudate ReHo values suggests that local neuronal activity becomes more disordered over time and the local connections of the neurons weakened. The caudate nucleus, which is the primary component of the basal ganglia, plays a crucial role in the brain's learning and memory system^11^. The hippocampus and caudate nuclei exhibited structural impairments in both adult and pediatric OSA patients^12^, Reduced brain metabolites, including *N*-acetyl aspartate and choline also appear in these regions in adult and pediatric OSA^12,13^ These results further confirm our finding.

Previous findings have demonstrated regional neuronal cell loss, along with significant morphologic and metabolic changes in untreated OSA patients which are attributed to hypoxia and neuro-inflammatory responses^12–14^. After CPAP treatment, our study indicated a change in ReHo of the caudate nucleus, suggesting that the function of this region was restored following after CPAP treatment. Previous research has suggested that long-term sleep fragmentation can result in a reduction of the caudate nucleus and orbitofrontal grey matter^15,16^. However, no change was observed in the recruitment of the caudate nucleus after sleep therapy. This may further suggest that the correction of hypoxia after CPAP therapy has a significant effect on the caudate nucleus function in OSA patients. Even a single night of CPAP treatment could potentially improve the caudate nucleus function.

In a previous ReHo study of OSA patients^17^, differences were reported in the cerebellum and other regions, which did not align with our finding. The participants in their study were primarily from high-altitude population differing from ours. Furthermore, our patients were patients with severe OSA, and the mean AHI (71.0 ± 21.1) was much larger than their study AHI (28.89 ± 20.44). Additionally, our statistical threshold was set to 0.01, lower than their study’s threshold of 0.05.

Compared to the HC groups, the fALFF signal in the bilateral cerebellar 8 region was increased in OSA patients. The correlation analysis also showed a significant negative correlation between cerebellar and blood oxygen saturation and the AHI. These results showed that OSA patients had significant effects on cerebellar function. Although our previous study^10^ reported that patients with OSA primarily suffer from intermittent hypoxia and sleep fragmentation, the correlation analysis in the current study suggests that hypoxia may have a more substantial effect on the cerebellum. Pae et al.^18^ reported that short-term intermittent hypoxia exposure elicits dose-dependent damage to cerebellar Purkinje and fastigial neurons. Chiu et al.^19^ recognized that intermittent hypoxia-induced oxidative stress on cerebellar astrocytes leads to cell loss in the cerebellum, which contributes to the dysfunction of the cerebellum. Park et al.^20^ found that OSA affects mainly the cerebellar pathway and that dysfunction in the cerebellum is associated with sleep fragmentation and hypoxia during sleep. These finding further support our results.

Our study found that the consistency of ReHo in the Frontal_Sup_R brain region decreased after CPAP treatment, and the fALFF values in the Frontal_Mid_Orb_L, Frontal_Inf_Orb_R, Frontal_Sup_Medial_L and other brain regions also changed after CPAP treatment. Further correlation analysis suggested a relationship between changes in fALFF values in the frontal lobes and alterations in REM sleep duration.

Previous studies has suggested that the frontal lobe serves as the emotional regulation center and has a significant relationship with cognitive function^21^. During REM sleep, emotional memories are consolidated in the prefrontal cortex^22^. REM sleep is associated with pyramidal neurons in the prefrontal cortex^23^. and its primary theta oscillatory feature in the frontal lobe is associated with the processing of recent emotional memories^24^. An increase in theta bands in the frontal lobe during sleep has also been reported in healthy participant by EEG and magnetoencephalogram analysis^25^. Due to the significant increase in REM time after CPAP treatment, the recovery of frontal lobe function further demonstrates the neuroimaging basis for cognitive function restoration Therefore, we believe that frontal lobe dysfunction is very common in patients with OSA and that the frontal lobe function undergoes changes after a single night of CPAP treatment. The frontal lobe serves an important cognitive and emotional center and an essential component of the default networks^26^. Numerous previous studies have suggested that patients with OSA may have changes in frontal lobe function and frontal lobe dysfunction^27–29^. It is well known that intermittent hypoxia and sleep fragmentation in OSA patients can lead to daytime sleepiness and cognitive dysfunction^30^. Daytime sleepiness in OSA patients is alleviated after CPAP treatment and short-term memory and reactivity can also be significantly improved.

Our study proposes that changes in frontal lobe function may serve as the neuroimaging basis for cognitive impairment changes. A study of cognitive function in moderate and severe OSA patients using ReHo also suggested that frontal lobe dysfunction in OSA patients could be a pathological basis for cognitive impairment^31^. Previously, only ALFF and ReHo had been reported in relation to CPAP, with no mention of fALFF^9,10^. However, studies on ReHo have also found changes in ReHo values in the frontal lobe, with one brain region being consistent with our findings^9^. In our earlier research^10^ utilizing ALFF, we identified variations in the insular, caudate, and calcarine lobes, but none in the frontal lobe. The possible reason is that fALFF is evaluated differently from ALFF, and our previous ALFF study was a frequency segmentation study, which may lead to inconsistent results.

The present study has notable design limitations. Firstly, the research focused solely on patients with severe OSA were studied, excluding women and those with mild to moderate OSA. Secondly, the sample sizes for both cases and controls were relatively small, which restricted the study’s statistical power. Thirdly, the absence of sham-CPAP therapy in the control group further limited the data analysis outcomes. Consequently, the findings of this study should be considered preliminary. In addition, the study only involved a one-night intervention for OSA patients and did not include any follow-up with them.

## Conclusion

The observation of abnormal fALFF and ReHo in OSA patients before and after a single night CPAP of treatment could enhance our understanding of the neurological mechanisms underlying severe OSA.

## Methods

### Subjects

A total of 30 newly diagnosed, untreated male OSA patients and 19 male HCs were recruited from the Sleep Medicine Center of West China Hospital, Sichuan University. The inclusion criterion of patients was an AHI > 30 events/h, and aged between 20 and 60. Exclusion criteria included a history of respiratory, neuropsychiatric or neurological disorders, alcohol, substance abuse or psychoactive medications. The inclusion criterion for the HC: AHI < 5 events/h, and aged between 20 and 60. None of the participants exhibited any health issues, as assessed by the clinic.

All participants provided written informed consent before undergoing MRI scans and other data acquisition. All methods were performed in accordance with the relevant guidelines and regulations, which was approved by the Human Research Ethics Committee of West China Hospital, Sichuan University (No.2019-09).

### Polysomnography

Overnight laboratory PSG recordings were acquired using a digital PSG recorder (Alice 6, Respironics, Orlando, FL, USA). The following variables were recorded: electroencephalography-, electromyogram -, electrooculography-, electrocardiogram, the oral and nasal airflow, snoring, thoracic and abdomen breathing movement, oxygen saturation (SaO_2_) and body position. The American Academy of Sleep Medicine^32^ rules were used to determine sleep staging, arousal and respiratory events. The polysomnography was monitored from 22:30 to 6:30 the next morning.

### Continuous positive airway pressure (CPAP) treatment

Following the PSG evaluation, a single night of CPAP treatment (22:30–6:30) was initiated. The device monitored breathing levels and automatically adjusted pressure. The ventilator’s treatment pressure was set at 4–20 cm H_2_O.

### fMRI data acquisition

All participants underwent functional and structural MRI imaging at our hospital using a 3.0 T MRI scanner (Siemens, Trio, Germany). The scan was performed the following day between 7:30 and 8:30 a.m. after the PSG or CPAP treatment. Rs-fMRI data were acquired using the following parameters: repetition time = 2000 ms, echo time = 30 ms, flip angle = 90°, thickness = 5.0 mm, gap = 0.5 mm, field of view = 240 × 240, matrix size = 64 × 64, and slices = 30; a total of 6000 rs-fMRI images were recorded. These s fMRI data acquisition steps have been previously described by our group^10^.

Before fMRI data preprocessing, we evaluated the imaging or head motion related artifacts of fMRI data. Prior to ReHo/fALFF analysis, DPARSFA (http://rfmri.org/DPARSF) and SPM12 (https://www.fifil.ion.ucl.ac.uk/) based on MATLAB2018b (Math Works, Natick, MA, USA) were used to preprocess the data: (1) Convert file format from DICOM to NIFTI; (2) Delete the first 10 volumes; (3) Slice timing and head motion correction; (4) T1 segmentation with the Diffeomorphic Anatomical Registration Through Exponentiated Lie algebra (DARTEL) spatial normalization into the MNI; (5) Regression of nuisance covariates including linear trend, white matter signals, cerebral spinal fluid signal, and Friston-24 parameters of head motions; (6) Smoothing with 6 mm full width at the semi-maximum Gaussian kernel(ReHo was first analyzed and smoothed, and fALFF were first smoothed).

### ReHo/fALFF analysis

ReHo and fALFF values were calculated using the DPABI software. ReHo was calculated for each subject by calculating the Kendall consistency coefficient of a given voxel time series and its nearest 26 voxels^8^. In the fALFF analysis, the time series of each voxel is transformed into the frequency domain to determine the power spectrum, and the sum of the amplitudes in the low-frequency range determines the fALFF. The fALFF maps for each participant were calculated as the proportion of the power spectrum of low frequency (0.01–0.08 Hz) across the entire frequency range^7^. Both ReHo and fALFF maps were then z-transformed for higher-level analyses.

### Statistical analysis

Statistical analyses were performed using SPSS software version 19.0 (IBM, Armonk, New York). Data normality was assessed using the (Shapiro–Wilk test), and Student *t* tests were performed to assess between group differences in demographic and sleep data that were normally distributed. The Mann–Whitney U-test was applied to assess between-group differences in variables that were not normally distributed. Continuous data are presented as mean ± SD for normally distributed variables and the median (P25, P75) for skew-distributed continuous variables.

We also conducted a statistical analysis of fALFF/ReHo using DPABI software. Whole-brain fALFF/ReHo comparisons were made between the pre-CPAP and HC groups using a two-sample *t* test, as well as between pre-CPAP and post-CPAP. Age, education level and head motion were imported as covariates. Multiple comparisons were corrected using the cluster-level GRF method. Different brain regions between pre- and post-CPAP were selected as ROIs. We extracted the value of each ROI with the center at the peak point and a radius of 5 mm for different brain regions. Subsequently, Pearson correlation analysis was performed on alteration of these ROIs and sleep data.

## Data Availability

The datasets used and/or analysed during the current study available from the corresponding author on reasonable request.
